# A machine-independent method to have active removal of 5,000 centistokes silicone oil using plastic infusion tube and 23-gauge microcannulas

**DOI:** 10.1186/s12886-015-0103-2

**Published:** 2015-08-25

**Authors:** Zhaotian Zhang, Yantao Wei, Xintong Jiang, Suo Qiu, Shaochong Zhang

**Affiliations:** State Key Laboratory of Ophthalmology, Zhongshan Ophthalmic Center, Sun Yat-sen University, 54S Xianlie Road, Guangzhou, 510060 China

## Abstract

**Background:**

To describe one modified method of having machine-independent removal of 5,000 centistokes silicone oil through 23-gauge trocar-cannulas.

**Methods:**

Consecutive patients with silicone oil tamponade for more than four months and with complete retinal reattachment were included. Two 23-gauge trocars were used to make sclerotomies while the microcannulas remained in situ for intravitreous infusion and silicone oil drainage. A short section of infusion tube was connected with a 10 ml syringe’s needle adapter. The other side was attached to the conjunctiva surface and covered the cannula’s cap inside to form a closed space for silicone oil drainage. The main outcomes were duration for complete removal of silicone oil and intra- and postoperative complications.

**Result:**

There were totally twenty cases (20 eyes) included. The mean time for draining out the silicone oil was 4.54 ± 0.78 minutes. Intraoperatively, flute needle was introduced additionally in seven cases to achieve complete removal. No cases experienced postoperative visual acuity deterioration or refractory hypotony. No significant residual oil bubbles were observed. No retinal redetachment occurred throughout the follow-ups.

**Conclusion:**

The modified method of using an infusion tube and 23-gauge trocar-cannulas can achieve quick and complete removal of high viscosity silicone oil.

**Electronic supplementary material:**

The online version of this article (doi:10.1186/s12886-015-0103-2) contains supplementary material, which is available to authorized users.

## Background

Silicone oil has been recognized effective for patients with complex retinal detachment to achieve reattachment. However, considering its high incidence of complications, it should be removed once the retina is completely reattached. One of the methods for silicone oil removal is anteriorly through the clear cornea incision by making a posterior capsulotomy in psuedophakic eyes or after cataract extraction [1]. It has the theoretical risks to damage structures in the anterior chamber, and limits intravitreous performance when necessary. Conventionally, we preferred the posterior approach via pars plana to have silicone oil removal. Larger sclerotomy may make quicker removal, but needs suturing and induces more surgical trauma and discomforts. Contrarily, smaller sclerotomy can be sutureless but difficult to have complete silicone oil removal, especially for silicone oil of high viscosity. To overcome this problem, we made modification of the sutureless method to be effective and safe, with the introduction of a common plastic infusion tube and two 23-gauge trocar-cannulas.

## Result

Using the current method, silicone oil could be completely removed in all the 20 cases. The average time for draining out the silicone oil was 4.54 ± 0.78 minutes (range from 3.0 to 6.0 minutes). Intraoperatively, flute needle was introduced additionally in seven cases to achieve complete silicone oil removal. Photocoagulation and epiretinal membrane peeling were performed in five and two cases, respectively. Sutures were needed in two cases. No intraoperative complications were observed. After the surgery, no cases experienced visual acuity deterioration or refractory hypotony. No significant residual oil bubbles were observed in the anterior chamber or in the fundus. Two cases complained of bubble floaters in front of the eye, but no secondary operation needed. There was no retinal redetachment occurred throughout the follow-ups.

## Discussion

The short plastic tube was an essential part to form a closed system including the vitreous cavity and the syringe, which was easily available in most ophthalmic institutions and low in cost. The whole system was easy to assemble within a few minutes. Additionally, machine-independence of our method makes silicone oil removal simple and economic. The modified method appeared to be widely applicable for many ophthalmic institutions, especially for those unequipped with vitrectomy system and could not perform machine-driven silicone oil removal.

With the development of transconjunctival sutureless vitrectomy system, there were efforts to use it for silicone oil removal [2–4]. Microcannula specially designed for silicone oil infusion and removal is now commercially available. The cannula needs to enter through the trocha’s microcannula into the vitreous cavity, thus narrowing the tunnel’s lumen [3]. This kind of technique could also lessen surgical trauma and decrease patients’ discomfort, but it is quite time consuming for eyes tamponaded with silicone oil of high viscosity. The current method can ideally overcome the problem and fully take advantages of the transconjunctival sutureless system. The metal cannula located through the sclera creates a really straight and firm tunnel permitting the silicone oil to be drained out.

Our data showed that the efficiency of the current method is equivalent with or higher than some previous reports [1–4]. The method is similar to the one reported by Song et al [4] in major procedures and materials. In the study, 5000 centistokes silicone oil was actively removed with the assistance of one section of tube linking a 23-gauge cannula to a machine. The mean time for the complete removal was 6.79 minutes. Although machine-independent, our methods also showed high efficiency. And there is no strict requirement of the plastic tube’s internal diameter to make gas-tightness. The internal diameter of the tube we used was somewhat larger than that of the cannula’s cap, but the flexibility of the conjunctiva beneath the tube is able to block the internal space from the external atmosphere.

The current method may have some disadvantages. Three factors may affect gas-tightness of the connection between the conjunctiva surface and the plastic tube. First, the patients’ conjunctiva should be flexible and flat enough; second, the plastic tube should also be flexible enough; and finally the plastic tube should be cut off carefully to form a flat transverse section. The sutureless sclerotomies may cause wound leakage, theoretically increasing the risk of postoperative endophthalmitis and hypotony.

## Conclusion

In conclusion, the introduction of a commonly used infusion tube is effective and safe for us to remove silicone oil of high viscosity in a sutureless and machine-independent approach. Controlled studies with larger samples are warranted to confirm the long term safety of the technique.

## Methods

### Patients

The study included 20 consecutive patients who were scheduled to have silicone oil (Oxane® 5700; Bausch & Lomb, Rochester, NY, US) removal between January 2014 and June 2014 (11 women and 9 men), with an average age of 51.7 years (range from 25 to 64 years). All the patients had silicone oil tamponade for more than four months, and had achieved complete retinal reattachment. All the surgeries were performed under local anesthesia by one experienced surgeon (S.Z.) at Zhongshan Ophthalmic Center of Sun Yat-sen University, Guangzhou, China. Informed consent was obtained from all the patients. All the patients were required to have postoperative follow ups for not less than six months. All the patients were informed of the planned surgical procedure. Written informed consent was obtained from all of the eligible subjects. The study was approved by the ethics committee of Zhongshan Ophthalmic Center (ZOC), and was conducted according to the tenets of the Declaration of Helsinki.

### Surgical technique

#### Step 1: Creation of transconjunctival sclerotomies with 23-gauge trocar-cannulas

Phacoemulsification and intraocular lens implantation were performed when the patients’ cataract was visual significant. 23-gauge trocar-cannulas (Alcon Lab., Inc., Fort Worth, TX) were used to create two transconjunctival sclerotomies at the inferotemporal and superotemporal quadrants. The trocars were withdrawn with the microcannulas in situ. The inferotemporal microcannula was for intravitreous infusion. And the superotemporal one was for silicone oil drainage. A bottle filled with balanced saline solution (Bausch & Lomb, Rochester, NY, US) was set at a height of 40 cm to generate an infusion pressure about 30 mmHg.

#### Step 2: Preparation of the plastic infusion tube and connection with the syringe

**Fig. 1:**
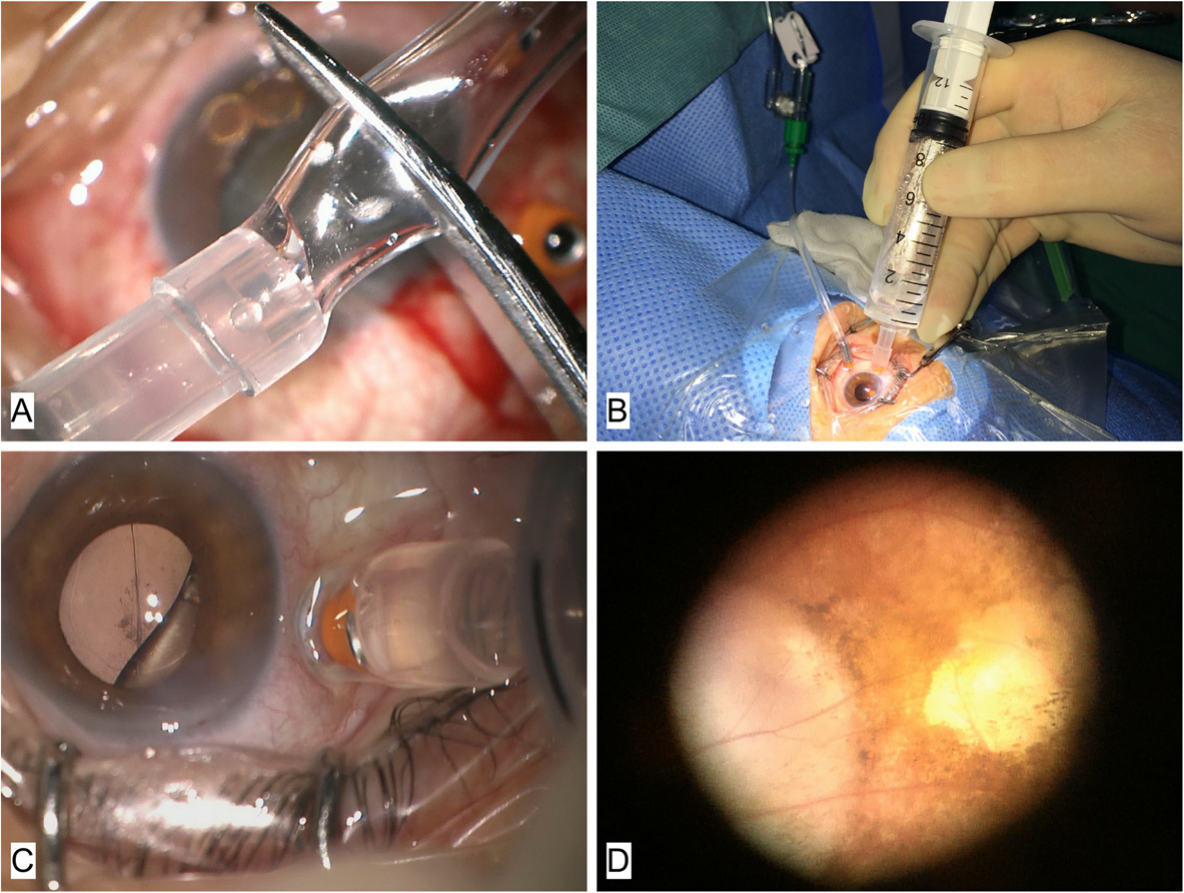
Main steps of the modified method. **a** After one end of the infusion tube is connected with the syringe’s needle adapter, a small segment about 6 mm is cut off transversely. **b** Infusion cannula is located at the inferotemporal quadrant. The syringe held by hand is attached to the conjunctiva surface covering the superotemporal microcannula’s cap inside to form a closed space. The hand of core plunger is fixed upwards by a vessel clamp. **c** Silicone oil is almost removed as the oil-fluid surface exists; the eye is rotated inferonasally to make the microcannula close to the eye’s apex. **d** Examination of the fundus to ensure no residual silicone oil and no intravitreous management needed

A commonly used plastic infusion tube was used. The internal diameter of which was about 3 mm. We cut off one small segment about 6 mm from it with scissors. After being slightly dilated by a vessel clamp, one end of the short tube was connected with the 10 ml syringe’s needle adapter (Fig. 1a).

#### Step 3: Attach the infusion tube to the conjunctiva surface and cover the microcannula to have active silicone oil removal

The other end of the tube was firmly attached to the conjunctiva surface, covering the microcannula’s cap inside to form a closed space from the external atmosphere. The core plunger is lifted manually and then fixed up by a vessel clamp snapping the handle’s inferior part, to generate a suction power on the silicone oil. Slight pressure vertical to the eye surface was added by the operator through the syringe, in order to improve gas-tightness of the attachment between the tube’s transverse section and the conjunctiva surface (Fig. 1b). Although the internal diameter is slightly larger than the external diameter of the microcannula’s cap, the soft conjunctiva beneath the plastic tube is able to block the internal space closed to the outer atmosphere.

#### Step 4: Slight adjustments during the procedure

By that, slight silicone oil inside the vitreous cavity was able to be smoothly drained into the syringe. The assistants recorded the time simultaneously. After the fluid-oil surface existed, the surgeon slightly rotated the eyes inferonasally making the microcannula for oil drainage closer to the eye’s apex, residual oil bubbles could be continuously drained into the syringe (Fig. 1c). After these procedures, silicone oil could be almost removed. Smaller oil droplets could be further drained out with assistance of a 23-gauge flute needle. Silicone oil bubbles in the anterior chamber could be cleared out by irrigation.

#### Step 5: thorough checking of the fundus

Wide angle viewing system or contact lens was used to check condition of the fundus (Fig. 1d). If retinal degeneration and epiretinal membrane existed, laser photocoagulation and membrane peeling were performed accordingly. After removal of microcannulas, absorbable sutures were placed to close the wounds with unstopped leakage. The whole surgical procedure was demonstrated in Additional file 1.

## Additional file

Additional file 1:
**Two 23-gauge trocar-cannulas were used to create sclerotomies at the temporal side of the eye.** The microcannulas are remained in situ for intravitreous infusion and silicone oil drainage. Patients with significant cataract are performed with routine phacoemulsification and intraocular lens implantation surgeries. After cataract surgery, the fundus becomes well visible. After one end of the infusion tube is connected with the syringe’s needle adapter, a small segment about 6 mm is cut off transversely. Infusion cannula is located at the inferotemporal quadrant. The syringe held by hand is attached to the conjunctiva surface covering the superotemporal microcannula’s cap inside to form a closed space. The hand of core plunger is fixed upwards by a vessel clamp. With negative pressure in the syringe, silicone oil is drained out quickly. Silicone oil is almost removed as the oil-fluid surface exists; the eye is rotated inferonasally to make the microcannula close to the eye’s apex. And finally, the surgeon has examination of the fundus to ensure no residual silicone oil and no intravitreous management needed. (MP4 15851 kb)
